# Association of nutritional support programs with zinc deficiency in Colombian children: a cross-sectional study

**DOI:** 10.1186/s40795-019-0305-8

**Published:** 2019-10-21

**Authors:** Ángela María Pinzón-Rondón, Alfonso Hoyos-Martínez, Daniela Parra-Correa, Ana María Pedraza-Flechas, Ángela María Ruiz-Sternberg

**Affiliations:** 1 Grupo de investigación clínica. Escuela de Medicina y Ciencias de la Salud, Universidad del Rosario sede Quinta de Mutis, Carrera 24 #6 3C-69, Bogotá, Colombia; 2 Department of Medical Education, Nicklaus Children’s Hospital, Miami, FL USA; 3 Grupo de Salud Pública. Escuela de Medicina y Ciencias de la Salud, Universidad del Rosario, Bogotá, Colombia

**Keywords:** Zinc, Child nutrition disorders, Food assistance, Income distribution, Colombia

## Abstract

**Background:**

Zinc is an essential trace element that plays a key role in the immune, gastrointestinal, respiratory and nervous systems*.* In Colombia, a vast percentage of children live in low-income households with food insecurity and nutritional deficiencies, including zinc. In an effort to improve children’s well-being, public health measures such as nutritional support programs that provide meals have targeted the poorest populations. The aim of the present study was to assess the role of nutritional support programs on zinc deficiency in Colombian children, while considering their wealth and food security.

**Methods:**

Cross-sectional study using data from the 2010 Colombian National Nutrition Survey, a population-based study representative of Colombia. A total of 4275 children between 12 and 59 months of age were included in the study. Stepwise logistic regressions were modelled with SPSS, first for zinc deficiency on wealth and food security, then adding enrolment in a nutritional support program, and finally, adjusting for socio-demographic variables.

**Results:**

A zinc deficiency prevalence of 49% was found. The adjusted models showed an association of wealth quintiles: very poor (OR = 1.48) and poor (OR = 1.39), food security (OR = 0.75) and enrolment in a nutritional support program (OR = 0.76) with zinc deficiency. Enrolment in nutritional programs did not modify the relationship of wealth and food security to zinc deficiency.

**Conclusion:**

Zinc deficiency is associated with wealth, food security and enrolment in nutritional support programs. Nutritional programs may be a good alternative against zinc deficiency, if they focus appropriately on the needs of children according to their wealth and food security.

## Background

Zinc is an essential trace element that is involved in over 400 enzymatic reactions and is present in more than 2000 proteins in the human body [1]. It plays a key role in the immune, gastrointestinal, respiratory and nervous systems, among others. Zinc has a critical function in gene expression, protein synthesis, cell development and replication, mainly in tissues that have a relatively high turnover rate [2, 3].

In children and adolescents, zinc deficiency could lead to growth retardation and stunting, developmental delays, impaired overall immune function and frequent infections, including respiratory infections, diarrhoea and malaria [4, 5]. It has been estimated that approximately 4% of the global burden of disease in children under 5 yrs of age is attributable to zinc deficiency. Furthermore, zinc supplementation has been shown to decrease childhood morbidity and mortality [6–9].

Compared to adults, children appear to have a higher zinc demand, which makes its deficiency more likely in this population [10, 11]. Zinc deficiency appears to be a widely spread problem for children under 5 yrs of age in developing countries. In China, its prevalence was estimated to be between 42 and 49%, which is similar to what has been reported in India [43.8%] and Colombia [43.3%] [12, 13, 15]. In Colombia, a two-fold increase from 20% in 2005 to 43.3% in 2010 has been reported [14, 15]. Although the burden of zinc deficiency was thought to be a health problem primarily in developing countries, in industrialized nations such as the United States, prevalence is as high as those seen in developing countries have been reported in underserved paediatric populations [16].

In Colombia, a vast number of children are exposed to poverty and food insecurity and to an environment that contributes to the incidence of a variety of nutritional problems, including zinc deficiency [17, 18]. In an effort to address this situation, the Colombian government has established several subsidized nutritional support programs for children [19], some of them are nationwide under the direction of the Colombian Institute of Family Wealthier (ICBF, from its initials in Spanish), and regionally there are also nutritional programs targeting children with low socioeconomical status. The national and regional programs include a nutritional supplement (Bienestarina), which provides 50% of the zinc daily recommended intake for this age group (1.5 mgs of zinc) [20, 21].

This study aims to study the role of nutritional support programs on zinc deficiency in Colombian under-five children while accounting for their wealth and food security.

## Methods

A descriptive, cross-sectional study with multivariate analysis, using data from the 2010 Colombian National Nutritional Survey (ENSIN, from its initials in Spanish), was designed.

### Data and sample

ENSIN 2010 was a joint effort of Colombian governmental and non-governmental organizations, which was supported by the United Nation’s World Food Program and the Pan-American Health Organization. The survey was applied to a nationally representative sample of 50,670 urban and rural households, which represent more than 99% of the Colombian population [15].

For this study, the initial sample included 4498 children, which comprised children from 12 to 59 months of age, who were included in the ENSIN. For the analysis, children with more than 10% of information missing in the survey (*n* = 223) were excluded from the analysis, for a final sample of 4275.

### Outcome measure

Trained bacteriologists went to the children’s houses, after signing an informed consent; they applied the surveys to the parents and collected the blood samples from the children, between 6 and 9 mL, by venepuncture of the median cubital vein. ENSIN determined the zinc levels using atomic absorption spectrophotometry (AA6300 Shimadzu) following the Colombian National Institute of Health standardized protocols [15]. For the purpose of the present study, zinc deficiency was recoded as a dichotomous variable, for which a serum level of less than 65 μg/dl on a non-fasting serum sample was deemed to be a deficient serum level (zinc deficiency, 1 = Yes and 0 = No).

### Independent variables

1) Self-reported information of enrolment in any nutritional support program, whether regional or national wide. This variable shows if a child is a beneficiary of a subsidized nutritional support program that provides at least one meal a day (one = Yes and 0 = No). 2) The wealth of the child’s household. This measure was created by the World Bank and Macro International to systematically determine a household’s relative economic status [19]. It gives each household a score based on a principal component analysis of the income, availability and quality of utilities, number of rooms, dwelling materials, type of cooking fuel, and availability of durable consumer goods. For the analysis, it was divided into quintiles (very rich, rich, average, poor and very poor). 3) Food security, which was assessed using the 2009 Latin-American and Caribbean household food security scale (ECLA), which is a validated scale based on household experiences [22]. For the analysis, food security was coded as a dichotomous variable (1 = Secure and 0 = Insecure).

### Control variables

The following control variables were included: ethnicity (recoded as a dummy variable, namely, Majority, Native-Colombian, Afro-Colombians and others), health coverage (1 = Yes and 0 = No), age in years, sex (1 = Girls and 0 = Boys), body mass index (BMI), maternal education level (recoded as a dummy variable, namely, Lack of education, Elementary, High school and Superior education), and area of residence (1 = Urban and 0 = Rural).

The serum vitamin A, ferritin, haemoglobin and C-reactive protein (CRP) levels and the weight and health status were included in the initial analysis but excluded from the final analysis because they failed to show any association.

### Data analysis

SPSS 22.0 (IBM) was used for the data processing. Initially, descriptive statistics were obtained, and logistic bivariate regressions were estimated for zinc deficiency on wealth, food security and enrolment in a nutritional support program. Finally, stepwise logistic multivariate regressions of zinc deficiency were performed. In the first models, wealth and food security were included, then enrolment in a nutritional support program was added, and finally, a complete model was computed by adjusting the previous models by all of the control variables.

Moderator analyses to find the possible effects of enrolment in nutritional programs on the associations of wealth and food security with zinc deficiency were conducted by multiplying the variables and introducing the terms into the regression models.

## Results

### Descriptive statistics

The final sample included a total of 4275 children with a mean age of 2.66 years (SD = ±1.09). Approximately half (49%) of the studied population had deficient serum zinc levels. A large percentage of the children (41.7%) belonged to the very poor category of the wealth quintiles, and most of the children (61.6%) attended a nutritional support program. Only 3.3% of the mothers lacked any form of formal education (see Table 1**)**.

**Table 1:** Descriptive statistics of studied variables

Variable	Total, *n*	%
	4275	
Enrollment in nutritional support program		
Yes	948	22.1
No	3327	77.8
Wealth		
Very rich	202	4.7
Rich	483	11.3
Average	735	17.2
Poor	1077	25.2
Very poor	1778	41.6
Food security		
Yes	1260	29.4
No	3015	70.6
Ethnicity		
Majority	3142	73.5
Native Colombians	549	12.8
Afro Colombians	543	12.7
Others	41	1.0
Health coverage		
Yes	3634	85.1
No	641	14.9
Age (years)	2.66 ± 1.09	
Sex		
Male	2274	53.2
Female	2001	46.8
Body mass index	0.95 ± 7.50	
Maternal education level		
Lack of education	151	3.5
Elementary	1465	34.3
High school	2134	49.9
Superior	525	12.3
Area		
Urban	2593	60.7
Rural	1682	39.3
Hemoglobin level (μg/dL)	12.87 ± 1.51	
Ferritin level (μg/dL)	33.01 ± 30.50	
Vitamin Alevel (μg/dL)	25.84 ± 8.86	
C reactive protein level (μg/dL)	0.41 ± 1.07	

SD, standard deviation

Bivariate regressions showed that enrolment in a nutritional support program (OR = 0.75), being poor (OR = 1.35) or being very poor (OR = 1.45) and having food security (OR = 0.65) were associated with zinc deficiency (see Table 2**).**

**Table 2:** Bivariate analysis for zinc deficiency vs. wealth, food security and enrollment in nutritional support programs

Variables	Exp (B)	Exp(B) 95% C.I.	
		Inferior	Superior
*Enrollment in nutritional support programs*	0,757	0,655	0,876
*Wealth*			
Very rich	Reference category		
Rich	0,862	0,618	1202
Average	1033	0,754	1413
Poor	1352	1005	1,83
Very poor	1453	1084	1948
*Food security*	0,651	0,530	0,800

*P* value < 0,05*

The multivariate analysis later revealed that even after adjusting for all of the control variables, the associations between enrolment in a nutritional support program (OR = 0.76), being poor (OR = 1.39), being very poor (OR = 1.48), or having food security (OR = 0.75) and zinc deficiency persisted. Additionally, an association between zinc deficiency and access to health services was found (OR = 0.87) (see Table 3**)**. Finally, no moderating effects of enrolment in nutritional programs on the association of wealth or food security with zinc deficiency were seen.

**Table 3:** Multivariate analysis adjusting for the control variables

Variables	Model 1				Model 2				Model 3			
	Exp (B)	EXP(B) 95% C.I.			Exp (B)	EXP(B) 95% C.I.			Exp (B)	EXP(B) 95% C.I.		
		Inferior	Superior			Inferior	Superior			Inferior	Superior	
*Enrollment in nutritional support program*					0,753	0,645	0,879	*	0,764	0,653	0,895	*
*Wealth*												
Very rich	Reference category											
Rich	0,862	0,618	1203		0,887	0,635	1237		0,887	0,634	1241	
Average	1027	0,750	1407		1083	0,790	1485		1086	0,789	1497	
Poor	1336	1001	1810	*	1423	1048	1930	*	1397	1014	1925	*
Very poor	1422	1060	1908	*	1534	1140	2064	*	1483	1067	2062	*
*Food security*	0,678	0,551	0,834	*	0,764	0,615	0,950	*	0,751	0,604	0,935	*
*Ethnicity*												
Majority	Reference category											
Indigene									1182	0,966	1448	
African descent									0,977	0,810	1180	
Others									1443	0,765	2720	
*Health coverage*									0,870	0,732	0,933	*
*Age*									1018	0,962	1076	
*Sex*									0,996	0,882	1125	
*BMI*									1002	0,994	1010	
*Education level of the mother*												
Superior	Reference category											
High School									1014	0,854	1203	
Elementary									0,904	0,746	1094	
Lack of education									0,855	0,585	1250	
*Area* (Urban)									0,933	0,801	1088	

*P* value < 0,05*

## Discussion

In this study, we found that zinc deficiency is associated with wealth, food security and enrolment in nutritional support programs. Alarmingly, almost half of the children under 5 yrs of age suffered from zinc deficiency in Colombia. This finding is comparable to what has been reported in other developing countries and underserved populations around the globe [12, 13, 15]. Considering the important role that zinc deficiency has on child morbidity and mortality, these results not only are worrisome but also highlight how zinc deficiency is a major public health problem in this population.

The negative association of enrolment in nutritional programs and zinc deficiency leads to the assumption that national programs to ensure child nutrition are probably protecting the children from further zinc deficiency and its catastrophic sequelae. Similar results have been found by other studies conducted in different countries, such as Mexico and Thailand, where programs that provide children with meals or fortified nutritional supplements showed a positive impact on serum zinc levels. [23, 24]

As expected, children developing under adverse conditions, such as poverty and nutritional insecurity, have increased risk for suffering of zinc deficiency. It thus follows that if a child is not able to adequately suffice his alimentary demands, he or she will likely develop nutritional problems, such as zinc deficiency [25]. An additional factor of dietary inadequacy in developing countries could be the prevalence of diets that tend to be plant-based, high in dietary fibre and phytic acid, and poor in animal protein, all of which limit zinc’s bioavailability [26–28].

Considering the evidence that has been provided, it is unsettling that such a large gap exists between the children who can access nutritional programs [22%) and those who are food insecure (71%). Further, the lack of moderation that the nutritional support programs exert over the association of zinc deficiency with poverty or food security suggests that the coverage is probably insufficient in these programs, as suggested by the fact that only 61.6% of the poorest population in this study was covered by the subsidy programs. Based on these results, efforts should be made to strengthen and expand the existing policies and to implement new ones that focus on these three areas to effectively reduce the zinc deficiency [29].

This study shows that poverty and food security are the determinants of zinc deficiency in Colombia. Previous studies have found associations of zinc deficiency and native ethnicity or household location in the Colombian population [30]. However, the full model shown in Table 3, reveals that the ethnicity and household location lose statistical significance when wealth and food security are included. Hence, it might be reasonable to assume that indigenous and rural populations in Colombia are more likely to be underprivileged and that poverty and food insecurity could be increasing the risk of dietary zinc inadequacy.

As has been widely described in the literature as well as in this study, access to health services is essential for a healthy childhood. Health coverage is a right for every child, regardless of his or her socio-economic background, and every measure directed to grant it should be taken [31].

Despite the great importance of recognizing Zinc deficiency, there is no unequivocal clinical or biochemical evidence of it and even though more than 32 biomarkers for Zinc deficiency had been described, none of them are considered reliable indicators of zinc status [32]. Zinc serum concentrations are difficult to measure adequately because they can be easily altered by external contamination when taking or processing the sample. However, although serum zinc concentrations are not useful for making individual diagnoses, they have been recommended as an indicator of population zinc status and can be used to assess the impact of supplementation programs at population level [33, 34].

Some of the strengths of this study are, its population-based nature and the availability of important socio-economical information such as, food security and household wealth. It is also important to emphasize that the cross-sectional nature of this study does not enable us to infer causality from these associations. Additionally, the independent variables are based on maternal report and therefore are subject to respondent bias. ENSIN failed to provide the exact nutritional supplementation that these children received, however this information was inferred from the national guidelines.

Another consideration is that the study is limited to Colombia. Although the results are likely to extrapolate to other countries with similar cultural and socioeconomic features, it’s important to keep in mind the singular characteristics within each country.

## Conclusion

Zinc deficiency is highly prevalent and a major public health problem in Colombia. It is positively associated with poverty and lack of food security. Subsidized nutritional support programs can alleviate zinc deficiency. To fight this problem, providing nutritional support through structured programs, mainly in areas with high poverty and food insecurity levels, could be an effective measure. All of the parts involved in the policy-making should make all efforts to strengthen and endure policies directed to improve these programs and should grant universal access to health services and reinforce nutritional security in the paediatric population.

## Data Availability

The datasets used and/or analysed during the current study are available from the corresponding author on reasonable request.

## Abbreviations

BMI: body mass index
CRP: C-reactive protein
ECLA: Latin-American and Caribbean household food security scale
ENSIN: Colombian National Nutritional Survey
ICBF: Colombian Institute of Family Wealthier
